# Exploring Antimicrobial Stewardship Influential Interventions on Improving Antibiotic Utilization in Outpatient and Inpatient Settings: A Systematic Review and Meta-Analysis

**DOI:** 10.3390/antibiotics11101306

**Published:** 2022-09-26

**Authors:** Ahmed A. Sadeq, Syed Shahzad Hasan, Noha AbouKhater, Barbara R. Conway, Abeer E. Abdelsalam, Jinan M. Shamseddine, Zahir Osman Eltahir Babiker, Emmanuel Fru Nsutebu, Stuart E. Bond, Mamoon A. Aldeyab

**Affiliations:** 1 Department of Pharmacy, Shaikh Shakhbout Medical City in Partnership with Mayo Clinic, Abu Dhabi P.O. Box 11001, United Arab Emirates; 2 Department of Pharmacy, School of Applied Sciences, University of Huddersfield, Huddersfield HD1 3DH, UK; 3 Department of Medicine, Shaikh Shakhbout Medical City in Partnership with Mayo Clinic, Abu Dhabi P.O. Box 11001, United Arab Emirates; 4 Institute of Skin Integrity and Infection Prevention, University of Huddersfield, Huddersfield HD1 3DH, UK; 5 Division of Infecious Diseases, Shaikh Shakhbout Medical City in Partnership with Mayo Clinic, Abu Dhabi P.O. Box 11001, United Arab Emirates; 6 Pharmacy Department, Mid Yorkshire Hospitals NHS Trust, Wakefield WF1 4DG, UK

**Keywords:** antimicrobial stewardship, interventions, antimicrobial use, multidisciplinary team, clinical practice

## Abstract

Antimicrobial stewardship interventions are targeted efforts by healthcare organizations to optimize antimicrobial use in clinical practice. The study aimed to explore effective interventions in improving antimicrobial use in hospitals. Literature was systemically searched for interventional studies through PubMed, CINAHL, and Scopus databases that were published in the period between January 2010 to April 2022. A random-effects model was used to pool and evaluate data from eligible studies that reported antimicrobial stewardship (AMS) interventions in outpatient and inpatient settings. Pooled estimates presented as proportions and standardized mean differences. Forty-eight articles were included in this review: 32 in inpatient and 16 in outpatient settings. Seventeen interventions have been identified, and eight outcomes have been targeted. AMS interventions improved clinical, microbiological, and cost outcomes in most studies. When comparing non-intervention with intervention groups using meta-analysis, there was an insignificant reduction in length of stay (MD: −0.99; 95% CI: −2.38, 0.39) and a significant reduction in antibiotics’ days of therapy (MD: −2.73; 95% CI: −3.92, −1.54). There were noticeable reductions in readmissions, mortality rates, and antibiotic prescriptions post antimicrobial stewardship multi-disciplinary team (AMS-MDT) interventions. Studies that involved a pharmacist as part of the AMS-MDT showed more significant improvement in measured outcomes than the studies that did not involve a pharmacist.

## 1. Introduction

In 2009, more than 3 million kg of antimicrobials were administered to humans in the US [1]. Despite the undeniable benefits of effective antimicrobial prescribing, there are significant risks associated with use and misuse, and antimicrobial resistance (AMR) is on the rise. Antimicrobial-associated *Clostridioides difficile* infection (CDI), adverse effects, and increasing antimicrobial and non-antimicrobial healthcare expenses are all major problems [2,3,4,5,6,7,8,9].

Although careful use of antimicrobial agents is widely recommended, their overuse or abuse has become entrenched in diverse contexts across the world [10,11]. AMR-related mortality is expected to exceed 10 million people per year by 2050 with improper antimicrobial usage now regarded as one of the major drivers of AMR [12,13,14].

Antimicrobial stewardship programs (ASPs) are targeted efforts by healthcare organizations or portions of organizations, e.g., inpatient (IP) settings, to optimize antimicrobial use, thus, improving patient outcomes, reducing negative consequences (such as AMR, or toxicity) and providing cost-effective therapy [3,15,16,17]. Such programs are multidisciplinary interventions that include patient-level stewardship (e.g., optimizing antimicrobial therapy for an individual patient based on culture results and clinical syndrome) and population-level stewardship (e.g., reducing overall antimicrobial consumption or consumption of a specific antimicrobial class through interventions) [10].

Between 20% and 50% of antimicrobial prescriptions in acute care hospitals are either unnecessary or inadequately administered [10]. Similarly, in outpatient (OP) settings, where the majority of antimicrobials are dispensed, misuse is unfortunately widespread. For instance, despite studies demonstrating that only 10% of people with sore throat have an antimicrobial-responsive illness, antimicrobials were prescribed for more than 60% of patients with pharyngitis [18]

One single systematic review (without meta-analysis) was published in Cochrane Library between January 2010 and April 2022 that investigated the impact of ASP interventions on improving antibiotic use in hospital settings. It concluded that those interventions have ensured that antibiotics were used more appropriately, the duration of antibiotic treatment was reduced, and length of hospital stay was decreased without increasing the risk of death [10]. By exploring other databases, we identified a few meta-analysis reviews within a similar period with objectives to improve antibiotic use, enhance clinical or microbiological outcomes, and/or decrease antibiotic treatment expenditure [19,20,21,22,23]. Three of those reviews focused on the outpatient setting while two involved inpatient care.

In the present meta-analysis, we have focused on reviewing clinical trials that investigated the impact of antimicrobial stewardship multidisciplinary team (AMS-MDT) interventions on improving clinical, microbiological, or other measured outcomes in two settings, outpatient and inpatient, in order to capture most of the interventions performed by antimicrobial stewardship teams in different clinical trials, and to differentiate effective interventions in each of the two settings.

The objective of this review was to identify antimicrobial stewardship program multi-disciplinary team (AMS-MDT) interventions and their impact on improving clinical and microbiological outcomes, and costs at a hospital level including inpatient and outpatient settings. We also aimed to identify the difference in the outcomes between studies that involved a pharmacist as a part of the AMS multidisciplinary team and those which did not involve a pharmacist.

## 2. Results

### 2.1. Search Results

**Figure 1:**
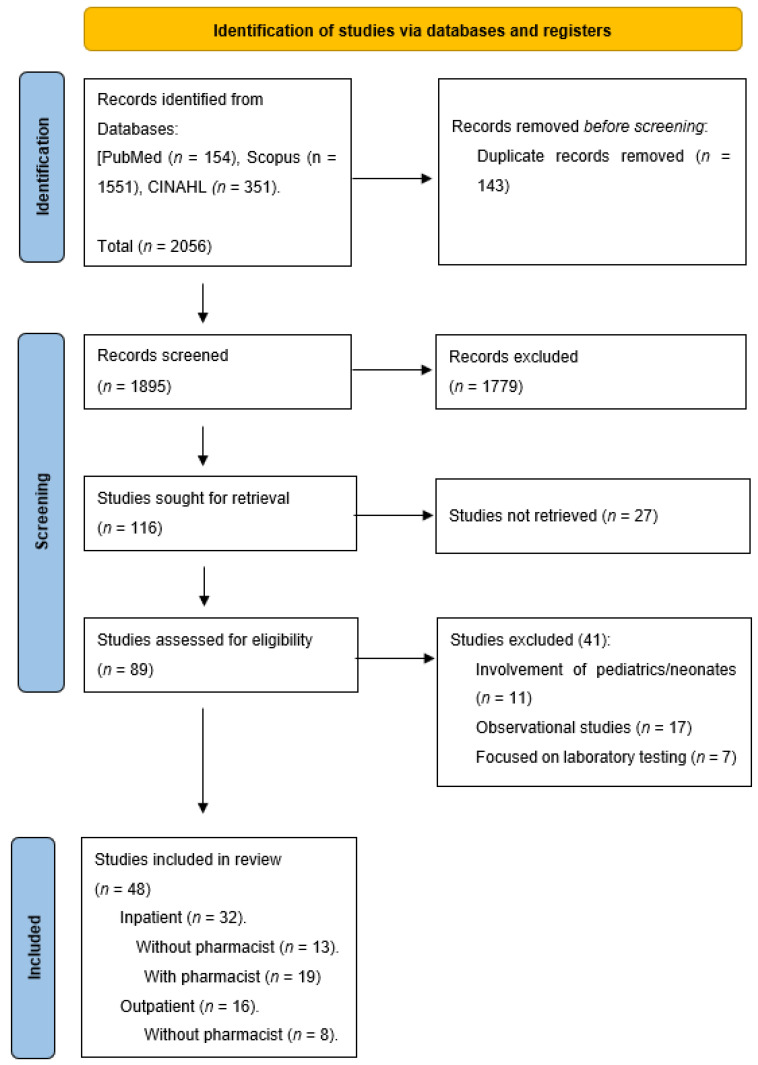
PRISMA flow diagram of the process of study selection.

A total of 2056 studies were generated by searching three databases: PubMed, Scopus, and CINAHL. Out of those, 1895 studies were screened by title, and out of those, 116 articles were sought for retrieval. After screening the abstracts for those articles, eighty-nine articles were fully retrieved, and by strictly implementing the inclusion and exclusion criteria, a final number of 48 articles were included in the study. Figure 1 represents the PRISMA (Preferred Reporting Items for Systematic Reviews and Meta-analyses) flow chart for this review. The included studies have been classified into four groups: 13 articles involved IP settings without a pharmacist as a part of an AMS-MDT intervention [24,25,26,27,28,29,30,31,32,33,34,35,36], 19 were carried out in IP settings with the inclusion of a pharmacist as a part of the intervention team [37,38,39,40,41,42,43,44,45,46,47,48,49,50,51,52,53,54,55], eight articles engaged OP without a pharmacist [56,57,58,59,60,61,62,63], while eight involved OP settings with a pharmacist [64,65,66,67,68,69,70,71]. Forty-one articles were from developed countries [24,25,26,27,28,30,31,32,34,35,36,37,38,39,41,42,43,44,46,48,50,51,52,54,55,56,57,58,59,60,61,62,63,64,65,66,67,68,69,70,71], while 7 took place in developing countries [29,33,40,45,47,49,53]. Table 1 represents the data extraction table. 

### 2.2. Quality Assessment

**Figure 2:**
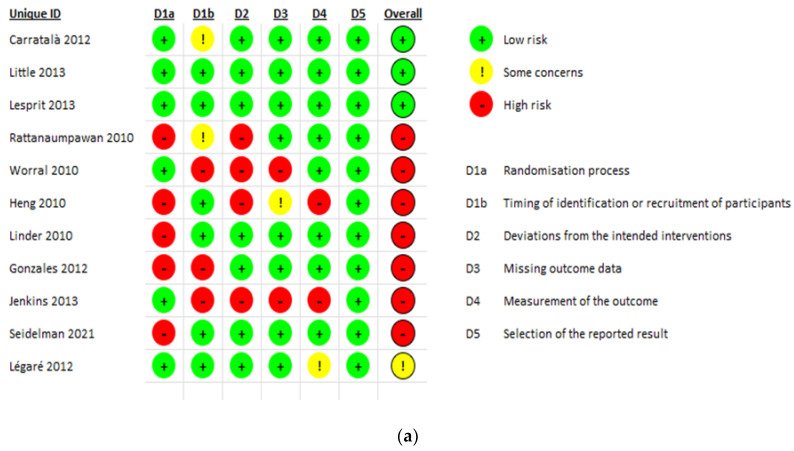

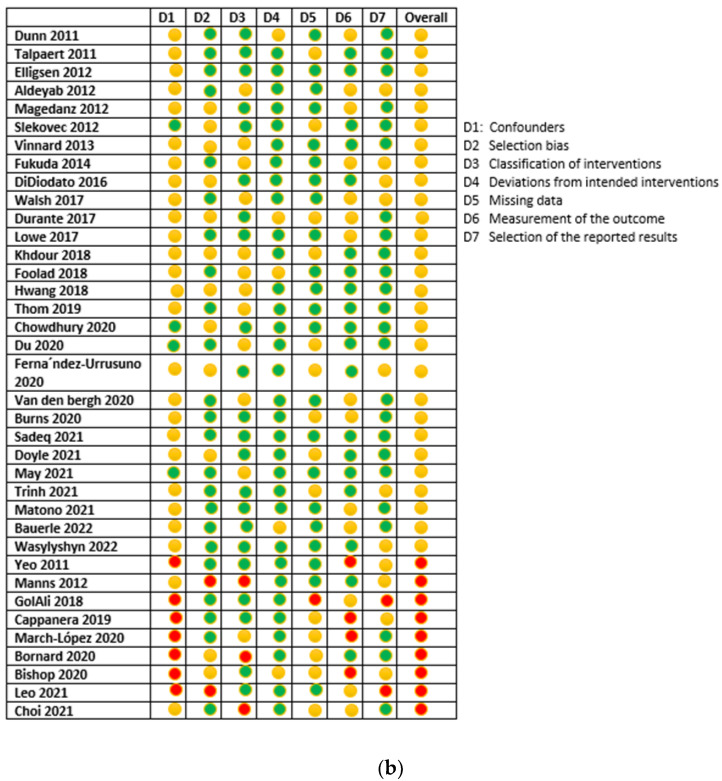
(**a**) RCTs risk of bias assessment using ROB-2 tool; (**b**) ROBIN-1 risk of bias assessment for non-randomized trials.

The risk of bias assessment for the included studies is presented in Figure 2. There were 11 RCT studies assessed using the ROB-2 assessment tool [28,32,33,34,46,57,58,59,60,63,67]. Of those, seven scored high risk [33,34,46,57,59,63,67], three scored low risk [28,32,60], with one article only found to have some concerns [58]. On the other hand, the remaining 37 articles were non-randomized before and after methodology and were assessed using the ROBINS-1 assessment tool [24,25,26,27,29,30,31,35,36,37,38,39,40,41,42,43,44,45,47,48,49,50,51,52,53,54,55,56,61,62,64,65,66,68,69,70,71]. Out of those 37, 29 were at moderate risk of bias [24,25,29,30,35,36,38,39,40,41,42,43,44,47,48,49,50,51,52,53,55,56,62,64,66,69,70,71] and 9 were at serious risk [26,27,31,37,40,45,54,61,68]. No article from those 37 scored low risks.

### 2.3. Interventions

There were 21 different interventions identified in the included articles as shown in Table 2. Of the identified interventions, twelve were captured in the IP setting [24,25,26,27,28,29,30,31,32,33,37,38,39,40,42,44,45,46,47,48,49,50,53,54,55] while 13 were in the OP setting [56,57,58,59,60,61,62,63,64,65,66,68,69,70,71], with 7 common interventions between the two settings. Those interventions were prospective and audit with direct intervention [37,38,42,44,45,47,48,49,50,53,54], education for health-care professionals (HCP) [25,28,40,43,56,66,68,69,71], antibiotic restriction and pre-authorization [24,30,33,61], use of clinical-decision support systems (CDSS) [31,39,46,66], regular dedicated infectious disease team (IDT) rounds [26,27,29,34], prospective audit and feedback [40,62], delayed antibiotic prescriptions [60,63], clinical decision making algorithm [36], intravenous (IV) to oral guidelines implementation [41], implementation of antibiotic time out [52], shared decision making [58], creating a quality dashboard [59], patient education [71], order sets implementation [69], developing clinical pathways for common OP infections [67], ASP multidisciplinary team escalating approach [55], Multi-faceted IDT visits (rounds, interactive training sessions, meetings) [26], retrospective audit and feedback [65,66], pocket cards containing antimicrobial guidelines [43], HCP education after audit and feedback [64], and soft stop orders [31].

### 2.4. Outcomes

Several common outcomes were identified in the included studies such as length of hospital stay (LOS), days of antibiotic therapy (DOT), 30-day readmission and mortality rate, antimicrobial guidelines’ adherence, CDI and multi-drug resistance (MDR) rates, antibiotic prescription rates, antibiotic consumption, defined daily dose (DDD), and cost-saving.

#### 2.4.1. Length of Hospital Stay

**Figure 3:**
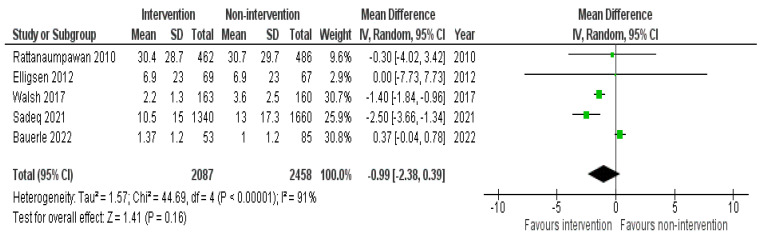
Pooled data from inpatient studies representing the impact of antimicrobial stewardship program intervention on length of hospital stay when comparing non-intervention with intervention groups.

LOS was significantly decreased in five studies that were conducted in the IP setting; three involved a pharmacist as part of an AMS-MDT [45,47,55] and two were without a pharmacist [28,36]. The other 6 studies reported insignificant results [25,32,33,36,37,53]. Interventions described in this meta-analysis were antibiotic restriction and pre-authorization, audit and feedback with direct intervention, clinical decision-making algorithm, HCP education, and ASP MDT escalating approach. The pooled effect size obtained using data from five studies failed to show a significant difference in the length of stay between the intervention and the non-intervention groups in the IP setting (−0.99; 95% CI: −2.38, 0.39) (Figure 3).

#### 2.4.2. Days of Therapy

**Figure 4:**
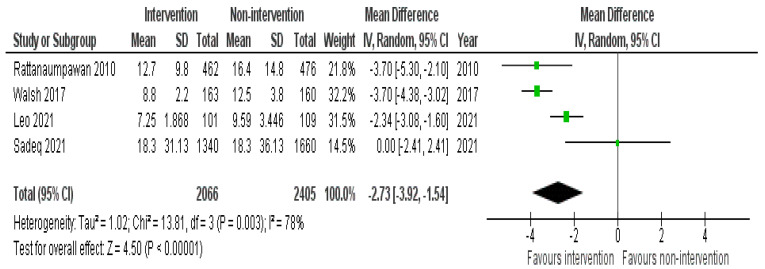
Pooled data from inpatient studies representing the impact of antimicrobial stewardship program intervention on days of antibiotic therapy when comparing non-intervention with intervention groups.

DOT was significantly reduced in 9 studies; out of those, four were in an IP setting without the involvement of a pharmacist [30,31,33,36], and four in an IP setting with the presence of a pharmacist [42,43,47,48]. One study was conducted in the OP setting and pharmacists did not take part in the intervention [62]. On the other hand, insignificant changes were reported by all the six studies conducted in the IP setting [29,34,35,38,52,55]. The data from four IP studies have been pooled to produce an overall effect. The overall pooled estimate was significant (−2.73; 95% CI: −3.92, −1.54) when comparing non-intervention with intervention group (Figure 4). Interventions that impacted DOT were HCP education, prospective audit and feedback with or without direct intervention, IV to oral guideline implementation, pocket cards containing antimicrobial guidelines, regular dedicated IDT rounds, and HCP education after audit and feedback.

#### 2.4.3. Thirty-Day Readmission and Mortality

**Figure 5:**
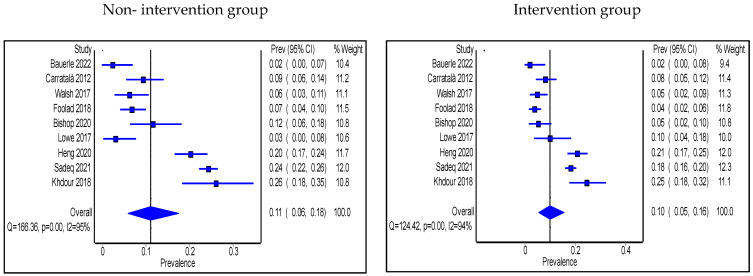
Prevalence of 30-day readmission within the inpatient setting in non-intervention and intervention groups.

**Figure 6:**
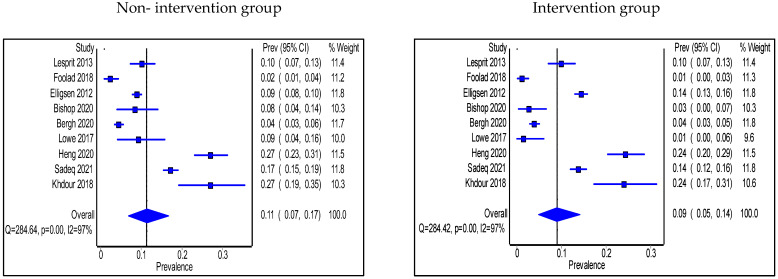
Prevalence of mortality within the inpatient setting in non-intervention and intervention groups.

Only two studies reported significant changes in 30-day readmissions [36,55] and both were conducted in the IP setting with one of them involving a pharmacist [36]. In contrast, eight studies found no significant differences [25,28,37,38,43,46,47,48]. Similarly, only three articles in the IP setting reported a significant reduction in mortality, with all of them having a pharmacist playing a role in the intervention [47,48,55], while no significant changes in mortality reported in the remaining studies [30,32,35,37,38,42,43,46,47,53]. The pooled proportion of patients who were re-admitted without AMS intervention was 11% (95% CI: 6%, 18%) and this was reduced to 10% (95% CI: 5%, 16%) with the intervention group as shown in Figure 5, while the pooled proportion of mortality was 11% (95% CI: 7%, 17%) in the non-intervention group compared to 9% (95% CI 5%, 14%) in the intervention group (Figure 6). Types of intervention that were used by the pooled studies with an impact on readmission and mortality were HCP education, clinical decision-making algorithm (only tested readmission), prospective audit and feedback with direct intervention, clinical-decision support system use, and MDT escalating approach. 

#### 2.4.4. Adherence to Antimicrobial Guidelines/Protocols

**Figure 7:**
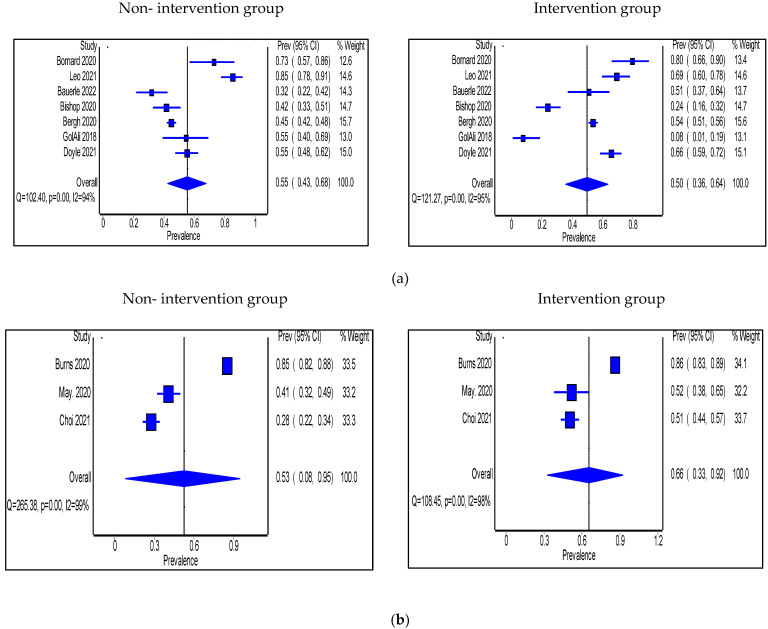
(**a**) Prevalence of patients prescribed antibiotics in accordance with hospital guidelines within the inpatient setting in the non-intervention and intervention groups; (**b**) Prevalence of patients prescribed antibiotics in accordance with hospital guidelines within the outpatient setting in the non-intervention and intervention groups.

Adherence to antimicrobial guidelines was significantly higher in the intervention group than in the non-intervention group (n = 7 studies); two in the IP setting had no pharmacist in the intervention [25,31], three in the IP setting included a pharmacist [37,45,53], and two studies were in the OP setting and involved a pharmacist [64,65]. No significant changes were seen in terms of adherence to guidelines in 2 studies where a pharmacist was engaged in the intervention, one was in the IP [39] and the other one was in the OP setting [69]. The pooled proportion of patients who were prescribed antibiotics in accordance with hospital antimicrobial guidelines in IP groups was 55% (95% CI: 43%, 68%) in the non-intervention group compared with 50% (95% CI: 39–64%) in the intervention group (Figure 7a). Types of intervention used were multi-faceted IDT round visits, pre-configured antibiotics, soft stop order, CDSS use, HCP education, and prospective audit and feedback with direct intervention. On the other hand, the pooled proportion of patients prescribed antibiotics as per the hospital antimicrobial guidelines in OP settings was 53% (95% CI: 8%, 95%) in the non-intervention group compared with 66% (95% CI: 33%, 92%) in the intervention group (Figure 7b), and the types of intervention used were HCP education after audit and feedback, HCP education, guidelines and order set implementations, and retrospective audit and feedback.

#### 2.4.5. Antimicrobial Use

**Figure 8:**
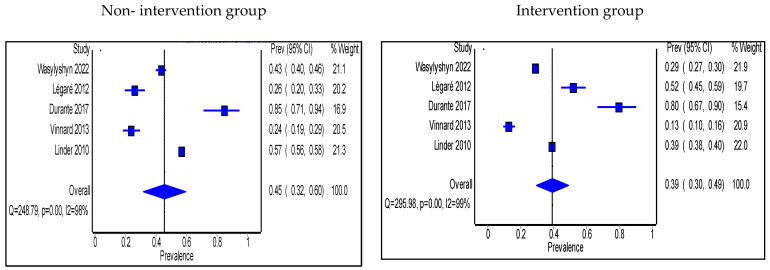
Prevalence of patients prescribed antibiotics in accordance with hospital guidelines within the outpatient setting in the non-intervention and intervention groups.

Antimicrobial use was expressed in two ways. First is the antimicrobial prescribing rate. Seven studies expressed antimicrobial use as prescribing rate and were all performed in OP settings [56,57,58,59,62,70,71]. Pharmacists took part in the intervention in only two of these studies [70,71]. The pooled proportion of patients prescribed antibiotics was 45% (95% CI: 32%, 60%) in the non-intervention group compared with 39% (95% CI: 30%, 49%) in the intervention group (Figure 8). Interventions that significantly decreased prescribing rate were shared decision making, prospective audit and feedback, HCP education, and antimicrobial treatment guidelines’ implementation. 

Daily defined dose (DDD) either alone, adjusted per patient days, or adjusted per inhabitant days, was the second measure used to express antimicrobial use, and was used in 11 studies. In the IP setting, three studies were without a pharmacist [27,29,33], and the results did not change significantly, while four out of six studies that engaged a pharmacist found a significant reduction [40,49,51,54]. In the OP setting, two studies involved a pharmacist as part of the intervention and concluded a significant reduction in one study [66] while the other did not report a *p*-value [68]. Interventions that significantly reduced DDD were antibiotic restriction and pre-authorization, prospective audit and feedback with or without direct intervention, HCP education, antimicrobial treatment guidelines’ implementation, retrospective audit and feedback, and CDSS use.

#### 2.4.6. Microbiological Outcomes

Microbiological outcomes were also measured in twelve of the included articles. Ten studies measured the difference in CDI rate between non-intervention and intervention groups; two were in the IP setting without the involvement of a pharmacist and reported a significant reduction in the rate [24,35], six in the IP setting with a pharmacist with 3 reporting a significant reduction [37,42,51]; the other three studies did not report significant changes in CDI rate [39,43,55]. Conversely, two studies investigated OP with a pharmacist and did not report significant changes [65,70]. Interventions that significantly decreased CDI rates were antibiotic restriction and pre-authorization, antimicrobial treatment guidelines’ implementation, and prospective audit and feedback with direct intervention.

Multidrug-resistant (MDR) organisms were investigated in two studies; one was in the IP setting [44] and resulted in a significant reduction in MDR rate, while the other was in OP setting [70] and reported insignificant changes. Both studies involved a pharmacist as a part of the intervention team. Interventions performed were prospective audit and feedback with direct intervention, HCP education, and prospective audit and feedback.

#### 2.4.7. Antimicrobial Therapy Cost

Cost-saving was analyzed in 5 studies; two of them were conducted in the IP setting without the presence of a pharmacist and three included a pharmacist in the intervention team and all of them reported a cost reduction [25,33,39,44,55]. Interventions conducted were HCP education, antibiotic restriction and pre-authorization, CDSS use, and MDT escalating approach.

### 2.5. Funnel Plots

To evaluate for publication bias, bias assessment in the form of funnel plots has been conducted (Figure S1–S4).

## 3. Discussion

Inappropriate use of antimicrobials could increase the development of AMR, necessitating the need for effective AMS interventions to optimize it [72,73]. Our review has summarized multi-disciplinary AMS interventions in two settings within hospitals, the outpatient and the inpatient settings. Our review has also been able to identify the interventions that have resulted in significant changes in the targeted outcomes. In addition, we have classified types of the intervention performed into four categories, i.e., inpatient with a pharmacist as a part of AMS-MDT intervention, inpatient without a pharmacist, outpatient with a pharmacist, and outpatient without a pharmacist. This allowed us to identify the impact of the presence of a pharmacist as a part of intervention in both outpatient and inpatient settings. Furthermore, the focus was on interventional studies to gain robust evidence with exclusion of any observational studies.

Length of hospital stay achieved significant reduction in more studies when a pharmacist was included in the intervention in the inpatient settings [45,47,55] compared to studies that did not involve a pharmacist. When data were pooled, AMS interventions resulted in lower LOS when compared with an opposing group without the intervention. This supports a previous study which proved that the implementation of hospital-based AMS reduced LOS by 8.9% [21]. In addition, the number of studies that applied multi-disciplinary interventions and achieved significant reductions in DOT were more than those which did not [30,31,33,36,42,43,47,48,70], and pooled data analysis showed a reduction of 2.73 mean days when AMS-MDT interventions were applied. The significant reduction in DOT was also found in a previous study that implemented a pharmacist-led ASP [74].

A significant reduction in thirty-day readmission was not observed in most of our included studies when using AMS interventions. On the other hand, only studies that included a pharmacist as a part of the multi-disciplinary intervention team achieved a significant reduction in mortality [47,48,55]. No previous meta-analysis has been found in the literature investigating the impact of ASP on readmission. Meanwhile, mortality decreased significantly in IP studies that involved a pharmacist, a result which was also concluded by two previous studies [11,74]. 

Another major outcome used to measure the impact the stewardship intervention studies was adherence to antimicrobial guidelines. In our review, studies that involved a pharmacist as part of the AMS-MDT indicated significant improvement in guidelines adherence [37,45,53]. This result is in line with a systematic review that included 57 articles from both IP and OP settings, and showed that clinical-decision-support system intervention improved adherence to antimicrobial guidelines twice as much in the intervention group than the non-intervention group [75]. 

Antibiotic consumption, represented by DDD, was reduced significantly only in studies that involved a pharmacist as part of the AMS-MDT [40,49,51,54,66], while it did not change significantly in other studies not involving a pharmacist. Antibiotic cost was reduced in all five studies that investigated expenditure, with three of them involving a pharmacist in the intervention [39,44,55]. This outcome was consistent with the findings of two past meta-analysis articles [76,77]. 

CDI and MDRO rates showed significant improvement in multiple studies conducted in the IP setting, and more than half of those studies had a pharmacist as part of the implemented intervention. 

Our review has some limitations. Firstly, it included non-randomized trials in addition to randomized trials; this was due to the number of studies in the literature using non-randomized before and after designs. Secondly, classification of the site of infection or type of infectious disease was not possible because most studies either tested only respiratory tract infections or did not specify an infection site. Third, some outcomes have a few numbers of studies, and this is probably because we included only articles that fit our study’s inclusion criteria. Finally, publication bias should be considered while interpreting our results. 

## 4. Materials and Methods

### 4.1. Search Strategy

This systematic review and meta-analysis followed the guidelines of the PRISMA (Preferred Reporting Items for Systematic Reviews and Meta-Analyses) protocol, an evidence-based set of items for reporting systematic reviews and meta-analyses [78]. 

We systemically searched PubMed, CINAHL and Scopus databases for related articles that were published in the period between January 2010 to April 2022. All articles investigating the impact of AMS-MDT interventions in hospitals and primary care were included for screening and review. The search strategy followed PIO (Population, Intervention, and Outcome) model and the keywords chosen for the search strategy were: **P** (hospital OR hospitals OR inpatient OR inpatients OR outpatients OR outpatient OR primary care) AND **I** ((antibiotic stewardship) OR (antimicrobial stewardship) OR (antibacterial stewardship)) AND **O** (outcome OR outcomes OR use OR utilization OR implementation OR prescribing OR prescription OR consumption OR mortality OR hospital stay OR therapy days OR difficile OR MDR OR MRSA OR ESBL OR Appropriate OR infection OR infections). Table 3 illustrates the full search strategy.

### 4.2. Study Selection

Two independent investigators (A. A. Sadeq and S. S. Hasan) examined titles and abstracts appearing in the database results to find potentially suitable publications. Any disagreements (e.g., including different articles by the two investigators) between the two authors were resolved by discussion and consensus.

For a study to be eligible for further screening and retrieval, the title or the abstract should have indicated an AMS-MDT intervention process that affected one or more of the outcomes of interest. The inclusion criteria for articles to be included in our review included interventional studies (whether randomized or non-randomized) that were conducted in hospitals or primary health care centers and investigated the impact of AMS-MDT interventions on improving clinical and microbiological outcomes, and cost.

Observational studies and articles that involved children or infants, discharge practice, antimicrobial surgical prophylaxis, long-term and nursing home facilities, interventions using rapid diagnostic tests, infection control practice, antifungals or antivirals, interventions conducted by nurses, special populations (e.g., renal disease), and online stewardship programs were all excluded from the final review. Exclusion criteria did not omit studies with high risk of bias. All inclusion and exclusion criteria assessments were carried out by two reviewers (A. A. Sadeq and S. S. Hasan).

### 4.3. Classification of Outcomes

The selected articles for our review were discussed in detail by two reviewers (A. A. Sadeq, S. S. Hasan), then agreed upon independently and then by consensus. The outcomes of interest were classified as clinical outcomes (days of therapy [DOT], length of hospital stay [LOS], 30-day readmission rate and mortality rate), microbiological outcomes (multi-drug resistant organisms [MDRO] resistance rates and CDI rates) and other outcomes including antibiotic prescribing rates, antibiotic consumption, and cost. 

Days of therapy are the number of days in which a patient has received antibiotic therapy, while length hospital stay is the difference in days between patient hospital admission and discharge. 

### 4.4. Data Extraction Process

The primary investigators established a standard data extraction form using Microsoft Excel®. This data extraction sheet was divided into four tables: Inpatient settings (IP) with a pharmacist as part of the AMS-MDT, IP settings without a pharmacist, outpatient settings (OP) without a pharmacist, and OP settings with a pharmacist. The following data were gathered from the identified studies: author name, year, country, sample size, study design, infection site, intervention type, outcomes, and findings. Data extraction was undertaken by two investigators (A. A. Sadeq and N. AbouKhater).

### 4.5. Risk of Bias/Quality Assessment 

The risk of bias was assessed using Version 2 of Cochrane risk-of-bias tool (RoB2) for randomized control trials [79]. Based on the responses to the signaling questions, an algorithm generated a proposed judgment regarding the risk of bias resulting from each area as ’Low risk of bias’, ’High risk of bias’, or ‘Some concerns’. The overall risk of bias generally corresponds to the worst risk of bias in any of the domains. 

For non-randomized trials, Risk of Bias in Non-Randomized Studies of Interventions (ROBINS-1) was used for bias risk assessment [80]. The overall risk of bias was judged depending on the scoring of the criteria; if the risk of bias for all domains was low then the overall risk was low, if there is a low or moderate risk of bias for all domains then it is moderate, while if there was a serious risk of bias or critical risk of bias in at least one domain, then the overall risk of judgment was serious or critical, respectively. 

The process of risk of bias assessment was performed independently by two investigators (A. Sadeq and N. AbouKhater) and any disagreements were resolved by discussion and consensus.

### 4.6. Study Registration

This review has been recorded in PROSPERO (The International Prospective Register of Systematic Reviews) under the code CRD42022302431.

### 4.7. Statistical Analysis

RevMan® software version 5.4.1 and MetaXL software version 5.2 were used to conduct the analyses with random-effects model to pool and evaluate data from eligible studies that reported the same outcomes. Pooled estimates were represented as a forest plot with a 95 percent confidence interval (CI) range for risk differences and mean differences. The I_2_ statistic was used to look at heterogeneity as it calculates the percentage of overall variation that can be attributed to between-study heterogeneity. I_2_ values of 25%, >50%, and >75% refer, respectively, to low, substantial, and considerable degrees of heterogeneity. Funnel plots were generated using inverse variance methods to examine the publication bias.

## 5. Conclusions

The present review has identified influential antimicrobial stewardship multidisciplinary team interventions in both inpatient and outpatient settings. Twenty-one interventions have been recognized with the most common interventions being prospective audit and feedback with direct intervention, antibiotic restriction and pre-authorization, regular dedicated ID rounds, HCP education, use of clinical decision support system, antimicrobial guidelines implementation, and retrospective audit and feedback. The inclusion of a pharmacist as a part of the multidisciplinary team increased the chances of achieving statistically significant changes in the outcomes. 

Those interventions were able to improve clinical (LOS, DOT, guidelines’ adherence, morbidity and mortality, and antibiotic prescription rate), microbiological, and cost outcomes when AMS-MDT interventions were applied and compared to non-intervention groups. 

## Figures and Tables

**Table 1:** Data extraction table.

AMS-MDT Intervention in Inpatient Settings (Without Pharmacist)								
Author, Year, Country	Sample Size (Male %)	Age	Study Design	Infection Type	Intervention	Outcome	Findings	Risk of Bias Assessment
Aldeyab et al., 2012, Ireland [24]	Not specified	Not specified	Interventional Pre and Post study.	CDI	**Antibiotic restriction** Restriction of high-risk antibiotics (second generation cephalosporins, third generation cephalosporins, fluoroquinolones and clindamycin).	Change in level of use of high-risk and medium-risk antibiotics.	Change in level of use of antibiotics (SE). Coefficient −14.2 (5.2); *p* < 0.01 Change in trend of use of antibiotics (SE). Coefficient: 20.5 (0.26); *p* = 0.08.	Moderate
						Change in CDI rates.	Change in level of CDI rates (SE): Coefficient: −0.02 (0.021); *p* = 0.3 Change in trend of CDI rates (SE): Coefficient: −0.001 (0.001); *p* < 0.01.	
Bauerle et al., 2022, US [25]	Non-intervention: 85 (57.6); Intervention: 53 (47.2).	Mean age (SD): Non: 39.5 (15.8); Intervention: 35.5 (13.2).	Interventional Pre and Post study.	Intra-abdominal infection	**HCP education** Empiric antimicrobial treatment selection for adult patients presenting with appendicitis.	The proportion of patients receiving the correct antibiotic.	Number of patients (%) Non: 27 (31.8%); Intervention: 27 (50.9%); *p* = 0.03.	Moderate
						LOS in days.	Mean LOS (SD): Non: 1 (1.2); Intervention: 1.37 (1.2); *p* = 0.08.	
						30-day readmission	Non: 2 (2.34%); Intervention: 1 (1.9%); *p* = 0.86.	
						Total cost ($).	Non: 4815.97; Intervention: 1444.98.	
Bornard et al., 2020, France [26]	Non-intervention: 37 (78); Intervention: 44 (68).	Mean age (SD) Non: 62 (18); Intervention: 59 (19).	Interventional Pre and Post study.	Health-care acquired infections.	**Multi-faceted ID round visit** Systematic visit three times/week of an IDS. interactive training sessions, Once daily meeting between intensivist and bacteriologist to discuss microbiological results.	Quality of empiric antibiotic therapies.	The prevalence of patients with appropriate antibiotic prescriptions: Non: 27 patients (73%); Intervention: 35 patients (80%); *p* = 0.31, ITS: No sudden change in levels (*p* = 0.67) and linear trend (*p* = 0.055).	Serious
Cappanera et al., 2019, Italy [27]	Non: NA. Intervention: 92	Not mentioned	Interventional before and after study.	Not specified	**Daily ICU rounds by infectious disease physicians** **Prescription audit and feedback**.	Consumption of carbapenems expressed as DDD/100 BDU.	DDD/100 BDU Non: 32888; Intervention: 2922; *p* < 0.76	Critical
Chowdhury et al., 2020, India [29]	Non: 140 (68). Intervention: 140 (77).	Range: 17–82 y. Mean age: (SD): Both groups together: 47.61 (14.54).	Interventional before and after study.	Not specified	**ASP rounds in the ICU.**	Antimicrobial use.	DDD/100 PD: Non: 98.66 Intervention: 91.62; *p* = 0.749. DOT/1000 PD: Non: 561 Intervention: 463; *p* = 0.337.	Moderate
Hwang et al., 2018, South Korea [30]	Not mentioned.	Not mentioned.	Interventional Pre and Post study, ITS.	Any site	**Antibiotic restriction.**	Antibiotic use (as DOT/1000 PD).	1- General wards: Non: 1065.98; Intervention: 1103.71; Change in level 106.81 (95% CI 40.10, 173.51); *p* <0.01; Trend change −28.14 (95% CI −37.51, −18.78); *p* < 0.01). 2- ICU: Non: 3945.29; Intervention: 3313.13; Change in level −1032.02 (95% CI (−1476.93, −587.11); *p* < 0.01. Trend change −50 (95% CI −109.11, 9.11); *p* = 0.093.	Moderate
						Mortality among ICU patients.	Mean APACHE 2 score: Non: 17.5; Intervention: 20.8; Level change: coefficient −0.537; *p* = 0.766. Trend change: coefficient 0.404; *p* = 0.171.	
Leo et al., 2021, Germany [31]	Non: 109 (56); Intervention: 101 (60.5)	Mean (SD) Non: 66.9 (11.9); Intervention: 65.7 (11.7)	Interventional Pre and Post study.	LRTS	**Multi-faceted ASP intervention:** Pre-configured antibiotics. Soft Stop Order. Clinical decision support for DOT.	DOT.	Mean DOT (SD): Non: 9.59 (3.446); Intervention: 7.25 (1.868); *p* < 0.01.	Serious
Lesprit et al, 2013, France [32]	Non-intervention: 377 (62.9); Intervention: 376 (60.1)	Median IQR Non: 66 (53–78). Intervention: 67 (54–78)	RCT	RTIS, UTIs, SSTI, IAIs.	**Prospective audit and feedback with direct intervention.**	Guideline adherence.	Number of patients (%): Non: 39 (36%); Intervention: 70 (69%); *p* < 0.01.	High
						Mortality.	Non: 38 (10.1%); Intervention: 37 (9.8%); *p* = 0.91.	
Rattanaumpawan et al., 2010, Thailand [33]	Non-intervention: 486 (52.9); Intervention: 462 (53)	Mean (SD) Non: 62.1 (18.8) Intervention: 63.5 (18.2)	RCT	Any site	**Antibiotic restriction and pre-authorization.**	Favorable clinical outcomes.	Number of patients (%): Non: 294 (60.5); Intervention: 319 (68.95); *p* < 0.01.	High
Seidelman et al., 2021, US [34]	Non-intervention: 2353; Intervention group: 2330.	Mean age (SD). Non: 61 (15.9). Intervention: 61.3 (16)	Cross-over RCT	Any site	**Weekly dedicated antibiotic stewardship handshake rounds.**	Antibiotic consumption (as DOT).	Mean DOT (SD) Non: 16.4 (14.8); Intervention: 12.7 (9.8); *p* < 0.01	High
Trinh et al., 2021, US [35]	Non: 892 (60). Intervention: 1122 (60)	Median age (IQR): Both groups together: 56 (55–57).	Interventional before and after study, ITS.	Febrile neutropenia.	**Guidelines’ implementation.**	DOT per 1000 PD of a composite of broad-spectrum IV antibiotics commonly used for Febrile neutropenia.	DOT/1000 PD: Non: 704; Intervention: 664; *p* = 0.85 Level change coefficient (95% CI): −39.6 (−109, 29.9) Trend change coefficient (95% CI): 1.13 (−1.55, 3.80)	Moderate
Walsh et al., 2017, US [36]	Pre-intervention: 160 (51.3). Post-intervention: 163 (52.8).	Mean SD Non: 55.3 (19.2) Intervention: 52.6 (19.2)	Interventional, pre and post study.	SSTI	**Clinical decision-making algorithm.**	CDI rate (standardized to 1000 PD).	Level change coefficient (95% CI): 0.15 (−1.59, 1.90). Trend change coefficient (95% CI): −0.004 (−0.06, 0.05)	Moderate
						Mortality (standardized to 1000 PD).	Level change coefficient (95% CI): −1.54 (−3.45, 0.38); *p* = 0.11. Trend change coefficient (95% CI): 0.04 (−0.01, 0.09); *p* = 0.11.	
**AMS-MDT Intervention in Inpatient Settings (With Pharmacist)**								
**Author, Year, Country, Hospital Size**	**Sample Size (Male %).**	**Age**	**Study Design**	**Infection Type**	**Intervention**	**Outcome**	**Findings**	**Risk of Bias Assessment**
Bishop et al., 2020, USA [37]	Non: 120 (51). Post: 113 (46)	Median age (IQR) Non: 63 (49–75). Intervention: 64 (54–72),	Interventional before and after study.	CDI	**Prospective audit and feedback with direct intervention.**	Proportion of patients treated with guideline adherent definitive treatment regimens within 72 h of CDI diagnosis.	Non: 50 (42%); Intervention: 27 (58%); *p* = 0.02.	Serious
						LOS in days	Mean LOS: Non: 12; Intervention: 11; *p* = 0.99.	
						Mortality	Number of deceased patients (%): Non: 10 (8%); Intervention: 3 (3%); *p* = 0.41	
						30-day readmission.	Number of readmitted patients (%): Non: 14 (12%); Intervention: 6 (5%). *p* = 0.08.	
DiDiodato et al., 2016, Canada [38]	Non-intervention: 238; Intervention: 525.	Not mentioned	Interventional before and after study.	RTIs	**Prospective audit and feedback with direct feedback.**	LOS	Difference in LOS 11% (95% [CI], −9, 35).	Moderate
						30-day readmission.	Intervention: OR = 0.79 (95% CI, 0.49, 1.29). No significant difference.	
						DOT.	HR: 1.24 (95% CI 0.99, 1.56) No significant difference.	
						Mortality.	OR = 0.79 (95% CI, 0.49, 1.29) No significant difference	
Doyle et al., 2021; Canada [39]	Number of prescriptions Non: 176. Intervention: 192.	Not mentioned	Interventional before and after study.	Not specified	**Clinical decision support system** (spectrum® mobile app)	Appropriateness of antibiotic prescriptions.	Non: 97 (55.1%); Intervention: 126 (65.6%); *p* = 0.051.	Moderate
						Inpatient AMU in DDD/100 PD.	DDD/100 PD: Non: 5600; Intervention: 5190; Relative reduction: −12%; Slope of trend line −6.62 DDD/1000/month.	
						CDI rate.	Cases/Inhabitants: Non: 11 cases (6.3/100,000); Intervention: 8 cases (4.4/100,000); Relative reduction: −30%; Slope of trend line −0.30 cases/month	
						Cost saving.	$82,078 per year.	
Du et al., 2020, China [40]	Non: 883 (54.59); Intervention: 880 (55.0).	Mean age (SD) Non: 61.97 (15.75); Intervention: 62.17 (16.87).	Interventional before and after study, ITS.	IAIs.	**Multifaceted interventions** Daily ward round. Regular review of medical orders: Giving feedback on the department’s antimicrobial management indicators. Necessary patient counselling and education.	Intensity of antibiotic consumption (as DDDs/100 PD).	Trend change: Non: Coefficient = 0.35; *p* = 0.34; Intervention: Coefficient = −0.88; *p* = 0.01.	Moderate
						LOS	Mean LOS (trend change): Coefficient = 0.02, *p* = 0.69.	
Dunn et al., 2011, Ireland [41]	Phase 1: Pre: 47 (44.7); Post: 73 (46.6). Phase 2 (intervention): Pre: 44 (51.2); post: 72 (47.2)	Mean age: Phase 1: Pre: 65; Post: 74. Phase 2 (intervention): Pre: 62; post: 62.	Interventional before and after Study.	Not specified.	**Implementation of IV to oral guidelines** Application of stickers to the drug chart. Clinical pharmacists to encourage IV to PO switch.	The duration of intravenous antimicrobial therapy.	Median hours of IV antimicrobials. Phase 1: Pre: 80; Post: 88; *p* = 0.59 Phase 2: Pre: 96; Post: 72; *p* = 0.02	Moderate
						IV courses switched on appropriate day.	IV courses switched on appropriate day (%) Phase 1: Non: 56.7; Intervention: 50.6; *p* = 0.257. Phase 2: Non: 55.5; Intervention: 71.7; *p* = 0.017.	
Elligsen et al., 2012, Canada [42]	Non-intervention: 2358 (67); Post: 2339 (69)	Mean SD Non: 63.8 (16.9). Intervention 63.3 (17.9).	Interventional before and after study, ITS.	No infection was specified.	**Audit and Feedback with direct intervention.**	Broad-spectrum antibiotic use (as DOT per 1000 PD).	DOT/1000 PDs: Non: 644; Intervention: 504; *p* < 0.01. Change in trend: Non: slop 1.9 (SE 3.66); Intervention: slop 6.1 (SE 3.82).	Moderate
Foolad et al., 2018, US [43]	Non-intervention: 307 (47.6) Intervention: 293 (51.9)	Median IQR Pre: 67 (53–78.5) Post: 66 (54–80).	Interventional before and after study	LRTIs	**Multifaceted approach** Education through pocket cards containing antimicrobial guidelines. Prospective audit and feedback by the pharmacist with direct intervention regarding appropriate DOT	DOT	Median DOT (IQR): Non: 9 (7, 10); Intervention: 6 (5, 7); *p* < 0.01.	Moderate
						CDI rate	Non: 0; Intervention: 0.	
						30-day Readmission	Non: 21 (7.1%); Intervention: 11 (3.8); *p* = 0.075.	
						Mortality	Number of deceased patients (%): Non: 7 (2.3%). Intervention: 3 (1%); *p* = 0.233.	
Fukuda et al., 2014, Japan [44]	Non-intervention: 3025 (gender no mentioned). Intervention: 1427 (822)	Mean age (SD) Non: not mentioned. Intervention: 78.3	Interventional before and after study.	Not specified	**Prospective audit and feedback with direct intervention**	Antimicrobial cost saving (USD per 1000 patient days).	Cost as USD per 1000 patient days: Non: 6133.5; Intervention: 4555.0; Relative cost reduction: 25.8%; *p* < 0.01.	Moderate
						Number of antimicrobials used (as DDDs per 100 PD).	Non: 1387; Intervention: 1388; *p* = 0.96.	
						LOS in days.	Mean LOS: Non: 16.6; Intervention: 15.9; *p* = 0.09.	
						Monthly detection rate of MRSA (as per 1000 PD).	Non: 2.9; Intervention: 1.5	
						Monthly detection rate of ESBL (as per 1000 PD).	Non: 0.4; Intervention: 0.3; *p* = 0.38.	
GolAli et al., 2018, Iran [45]	Non: 44 (27) Intervention: 39 (19).	Mean age (SD) Non: 62.7 (17.3). Intervention: 64.6 (17.3).	Interventional before and after study.	Any infection site.	**Prospective audit and feedback with direct intervention.**	Appropriate-ness of antimicrobial consumption.	**Rate of discrepancies from guideline (number of patients):** Antibiotic choosing: Non 24 (54.54%); Intervention: 3 (7.69%); *p* < 0.01. Dosing schedule: Non: 19 (43.18%). Intervention: 5 (12.82%); *p* < 0.01. De-escalation: Non: 30 (68.18%); Intervention: 8 (20.51%); *p* < 0.01. Conversion to oral regimen Non: 33 (75%); Intervention: 6 (15.38%); *p* < 0.01	Serious
						LOS in days	Mean LOS: Non: 16.1. Intervention: 11.6. *p* < 0.01	
Heng et al., 2020, Singapore [46]	Non: 455 (59) Intervention: 416 (54).	Median age (IQR) Non: 74 (45 -93). Intervention: 76 (48–93).	RCT.	Not specified.	**CDSS (Compulsory vs. on-demand).** Provides guidance on antibiotic use and infection management based on hospital guidelines.	Mortality.	Number of deceased patients (%): Non: 123 (19%); Intervention: 102 (16); *p* = 0.22 (HR: 0.87, 95% CI 0.67, 1.12)	High
						30-day readmission.	Number of readmitted patients (%): Non: 92 (14%). Intervention: 87 (14%); *p* = 0.91.	
						LOS in days.	Median LOS (IQR): Non: 15 (5–64); Intervention: 15 (4–70); *p* = 0.92.	
Khdour et al., 2018, Palestine [47]	Non: 115 (47.8). Intervention: 142 (57.7).	Mean age (SD) Non: 68.4 (15.3). Intervention: 68.4 (15.3).	Interventional before and after study	Not specified.	**Prospective audit and feedback with direct intervention.**	Compliance with or rejection of ASP recommendations	Recommendations accepted: 138 Total recommendation: 176; Acceptance rate: 78.4%.	Moderate
						DOT.	Median DOT (IQR) Non: 11 (3–21); Intervention: 7 (4–19); *p* < 0.01.	
						LOS.	Median LOS (IOR): Non: 11 (3–21); Intervention: 7 (4–19); *p* = 0.01.	
						Mortality.	Number of deceased patients (%): Non: 31 (26.9%). Intervention: 34 (23.9%); *p* = 0.1.	
						30-day Readmission	Number of readmitted patients (%): Non: 30 (26.1%). Intervention: 35 (24.6%); *p* = 0.5.	
Lowe et. al., 2017, Canada [48]	Non: 98 (48); Intervention: 70 (30)	Mean age (SD) Non: 72 (23–103); Intervention: 70 (21–94).	Interventional before and after study.	RTIs	**Prospective audit and feedback with direct intervention** Based on 2 criteria: microbiology and chest imaging.	Duration of antimicrobial therapy after viral diagnosis (DOT).	Mean DOT (SD) Non: 4.1 (0–14); Intervention: 2.8 (0–12); Difference: −1.3 (95% CI −0.3, −2.3); *p* < 0.01.	Moderate
						LOS in days.	Mean LOS (range): Non: 9.6 (1–70) Intervention: 14.3 (1–92); *p* = 0.07	
Magedanz et al., 2012, Brazil [49]	Not mentioned	Not mentioned	Interventional before and after study.	Not specified	**Prospective audit and feedback with direct feedback**	Use of antibiotics (consumption) represented as DDD/100 PD).	DDD/100 PDs: Non: 48.9; Intervention: 36.9; *p* < 0.01. Change in level: Co-efficient: 4.69; *p* = 0.37 Change in trend: Co-efficient: 1.20; *p* = 0.004	Moderate
Matono et al., 2021, Japan [50]	Non: 59,195 Intervention: 3935.	Adult and neonates	Interventional before and after study, ITS	Not specified	**Prospective audit and feedback with direct intervention.**	Trend in monthly carbapenem consumption.	Co-efficient= −3.02; 95% CI: −4.63, −1.42, *p* < 0.01.	Moderate
Talpaert et al., 2011, UK [51]	Non: 380; Intervention: 247 Male% not mentioned	Not mentioned	Interventional before and after study, ITS	CDI	**Revised antibiotic guidelines.** **Development and implementation of antibiotic stewardship**	Change in the levels of targeted antibiotic consumption (as DDDs/1000 OBD).	Change in level (95% CI): 42.04 (−178.34, 262.42); *p* = 0.695 Change in trend (95% CI): −233.22 (265.94, 20.50); *p* = 0.047.	Moderate
						CDI rate.	CDI rate: Intervention: Decrease in CDI [incidence rate ratio (IRR) 0.34; 95% CI 0.20–0.58, *p* < 0.01]. CDI trend change (IRR, 95% CI): Non: 0.93 (0.88, 0.99), *p* = 0.015; Intervention: 1 (0.94, 1.06); *p* = 0.94.	
Thom et al., 2019, US [52]	Non: 1541. Intervention: 1929. (Gender not mentioned)	Median age 65 (44–80)	Interventional before and after study	Not specified	**Implementation of antibiotic timeout (ATO).** A provider-driven ATO on antibiotic days 3–5 was prompted by the care team on each unit during rounds without direction from research or stewardship teams.	DOT.	Mean DOT: Non: 12.7; Intervention: 12.2; *p* = 0.17.	Moderate
						Total antibiotic DOT (in hospital and at discharge) per patient admission.	Mean DOT: Non: 18.9; Intervention: 18.2; *p* = 0.67.	
						Reception of inappropriate antibiotics on antibiotic days 3–5.	OR: 0.58 (95% CI, 0.48, 0.69); Significant difference.	
Van der bergh et al., 2020, South Africa [53]	Non-intervention: 1247 (38.9); Intervention: 1217 (42.1)	Mean age: Non: 60; Intervention: 58.3.	Interventional before and after study.	CAP	**Prospective Audit and feedback with direct intervention.** Pharmacist interacting with physician to implement the newly developed CAP bundle guidelines.	CAP bundle compliance rates.	Number of patients (%): Non: 560 (47.3%); Intervention: 653 (53.6%); Difference: 5.8% (95% CI 4·1, 7·5); *p* < 0·01.	Moderate
Yeo et al., 2011, Singapore [54]	Non: not mentioned Intervention: 556	Not mentioned	Interventional before and after study, ITS.	Not specified	**Prospective audit and feedback with direct feedback**	Trend of DDD/100 PD of audited antibiotics.	Non: DDD/100 PD: 46.12; Trend coefficient 0.019, *p* = 0.98; Intervention: DDD/100 PD: 52.71; Trend coefficient −2.5, *p* = 0.001.	Serious
Sadeq et al., 2021, UAE [55]	Non: 1660 (71); Intervention: 1340 (59)	Mean age (SD) Non: 54 (18.6); Intervention: 60 (21)	Interventional before and after study.	Not specified	**Escalating approach involving Prospective audit and feedback with direct intervention.**	LOS in days	Mean LOS (SD) Non: 13 (17.3); Intervention: 10.5 (15); *p* < 0.01	Moderate
						DOT	Mean DOT (SD) Non: 18.3 (36.13) Intervention: 18.3 (31.13); *p* = 0.2.	
						30-day readmission	Number of readmitted patients (%): Non: 403 (24) Intervention: 244 (18) *p* < 0.01.	
						Mortality	Non: 285 (17); Intervention: 184 (14); *p* < 0.01.	
						CDI	Non: 0 cases; Intervention: 6 cases.	
**AMS-MDT Intervention in Outpatient Settings (Without Pharmacist)**								
**Author, Year, Country, Hospital Size**	**Sample Size (Male %).**	**Age**	**Study Design**	**Infection Type**	**Intervention**	**Outcome**	**Findings**	**Risk of Bias Assessment**
Durante et al., 2017, US [56]	Non: 39. Intervention: 49.	Mean age Non: 51.5. Intervention: 49.8.	Interventional before and after study.	RTS	**Provider education** Through “lunch-and-learn” presentation session.	Reduction of antibiotic prescriptions.	Number of patients received antibiotics (%): Non: 33 (84.6%). Intervention: 39 (79.2%).	Moderate
Gonzales et al., 2013, US [57]	Control: 4145 (1782). Baseline: 3195 (1396). Study: 950 (386) PDS: 4640 (1849) Baseline: 3639 (1470). Study: 1001 (379). CDS: 3991 (1610) Baseline: 2974 (1225). Study: 1017 (385)	13–64 y.	RCT	RTIs	**HCP education:** Patient decision support (PDS): Through a print-based strategy. Computerized Decision support (CDS): Through an electronic medical record-based strategy.	Percentage of patients prescribed antibiotics for uncomplicated acute bronchitis.	**Percentage of patients (%):** Control: Baseline: 3005 (72.5%). Study: 3080 (74.3%). PDS: Baseline: 2911 (80%) Study: 684 (68.3%). CDS: Baseline: 2201 (74%). Study: 977 (60.7%). Control vs. PDS: *p* = 0.003; Control vs. CDS: *p* = 0.014. PDS vs. CDS: *p* = 0.67.	High
Légaré et al., 2012, Canada [58]	Control group: Pre-intervention period: 169 (68); Intervention period: 180 (62) Intervention group Pre-intervention period: 178 (57). Intervention period: 181 (64).	Mean age (SD) Control group: Pre-intervention: 43.3 (16.2) Post-intervention: 39.3 (12.4) Intervention group Pre-intervention: 43.3 (14.8). Post-intervention: 40.8 (15.1)	RCT	RTIs	**Shared decision-making** The online tutorial addressed key components of the clinical decision-making process about antibiotic treatment for acute respiratory infections in primary care.	The proportion of patients who decided to use antibiotics immediately after consultation.	Intervention group Non: 46 (27.2%); Intervention: 94 (52.2%); ARR: 0.5 (95% CI 0.3, 0.7).	Some concerns
Linder et al., 2010; Spain [59]	Non: Patients:73,826 (27,399). RTI Visits: 10,082. Intervention: Patients 62,807 (22,053). RTI Visits: 8406.	Mean age (SD) Non: 49 (17). Intervention: 49 (17).	RCT	RTIs	**Quality Dashboard** [An electronic health record (HER)-based feedback system].	Antibiotic prescribing rates.	Number of RTIs patients’ sits (%): Non-intervention: 4761 (47%); Intervention: 3912 (47%); *p* = 0.87.	High risk
Little et al., 2010, UK [60]	309 non-pregnant women randomized to five groups.	18–70 Y	RCT	UTI	**Multifaceted approach** empirical delayed (by 48 h) antibiotics. Targeted antibiotics based on a symptom score. Dipstick result Positive result on midstream urine analysis.	Symptom severity (days 2 to 4).	**Mean frequency symptom severity score (mean difference with 95% CI)** Immediate antibiotics (as control group) 2.15 (SD 1.18). Midstream urine: 2.08 (−0.07; −0.51, 0.37). Dipstick: 1.74 (−0.40; −0.85, 0.04). Targeted antibiotics based on symptom score: 1.77 (−0.38; −0.79. 0.04). Delayed antibiotics 2.11 (−0.04; −0.47, 0.40). *p* = 0.177.	Low
Manns et al., 2012., Canada [61]	170,247 (42.7)	Median age IQR 74 (69, 80)	Interventional before and after study, ITS	UTIs and RTIs.	**Optional special authorization program** Restricting the use of quinolones to defined subgroups of patients with common outpatient infections.	Use of a quinolone within the 30 day period following a unique index visit for UTI and RTIs.	Level change: −3.5 (95% CI −5.5, 1.4) prescriptions per 1000 index visits. *p* = 0.74.	Serious
Wasylyshyn et al., 2022, US [62]	Non: 972 (26.7) Intervention: 3562 (30.2).	Mean age: Pre: 49 Post 44.	Interventional before and after study.	RTIs	**Multifaceted interventions:** 1- Prospective audit and feedback. 2- Guidelines development. 3- Using questionnaire to support gathering pertinent information to provide nudges for guideline-concordant prescribing	Rate of antibiotic prescribing.	Number of patients (%): Non: 420 (43.2%); Intervention: 1028 (28.9%); *p* < 0.01.	Moderate
						Mean DOT	Non: 10 days; Intervention: 5 days; *p* < 0.01	
Worral et al., 2010, Canada [63]	Number of prescriptions (patients) Usual (control): 74. Post-dated: 75.	≥18 y.	RCT	URTS	**Delayed antibiotic prescriptions** (2 days later)	Whether or not the prescriptions were filled.	Number of filled prescriptions (%): Usual prescriptions: 32 (43.2%); Post-dated prescriptions: 33 (44.0%); *p* = 0.924.	High
						The time it took for the patients to fill the prescriptions.	Number of prescriptions filled early (%): Usual: 16 (50%); Post-dated: 16 (48%); *p* = 0.975. The time it took to fill the other 33 prescriptions (in days): Usual: 6.1; Post-dated: 6.5; *p* ≤ 0.968.	
**AMS-MDT Intervention in Outpatient Settings (With Pharmacist)**								
**Author, Year, Country, Hospital Size**	**Sample Size**	**Age**	**Study Design**	**Infection Type**	**Intervention**	**Outcome**	**Findings**	**Risk of Bias Assessment**
Burns et al., 2020, US [64]	Number of prescriptions: Non-intervention: (30 RTI,20 UTI) = 50 Intervention: (825 RTI, 282 UTI) = 1107	Not mentioned.	Interventional before and after study.	RTIs and UTIs.	**HCP education after audit and feedback** Education and guidelines were provided before the intervention period.	1- Rate of compliance to antibiotic prescribing guidelines. 2- Proportion of Prescriptions with appropriate duration	For UTIs: Number of prescription compliant to the Guidelines (%): Non: 4 (20%) Intervention: 195 (69.2%). Number of prescription compliant to the Guidelines (%): Non: 13 (43.3%). Intervention: 716 (86.8%). For RTIs: Number of prescription compliant to the Guidelines (%): Non: 4 (20%) Intervention: 195 (69.2%). Number of prescriptions with appropriate duration (%): Non: 18 (60%). Intervention: 687 (83.3%). Total compliance rate: Non: 432/506 = 85%; Intervention: 480/558 = 86%.	Moderate
Choi et al., 2021, US [65]	Non-intervention: 200 (18.5) Intervention: 200 (23).	Mean age (SD) Non: 56 (19). Intervention: 57 (18).	Interventional before and after study.	UTIs and SSTIs.	**Retrospective audit and feedback.**	Total antibiotic regimen appropriateness.	Number of patients with appropriate antibiotic prescriptions (%): Non: 55 (27%); Intervention: 101 (50%); *p* < 0.01.	Serious
						CDI rate	Non: 0; Intervention: 0; *p* = 0.99.	
Ferna’ndez-Urrusuno et al., 2020, Spain [66]	Not mentioned.	Not mentioned.	Interventional before and after study, ITS.	Not specified.	**Multi-faceted intervention.** Development of electronic decision support tools Local training meetings. Regional workshops. and conferences. Targets for rates of antibiotic prescribing linked to financial incentives. Feedback on antibiotic prescribing. Implementation of a structured educational ASP.	Rates of antibiotics use [as DDD per 1000 inhabitants-day (DID)].	Trend change: Non: 0.19 (95% CI 0.13, 0.25); *p* < 0.01. Intervention: −0.71 [−0.84- (−0.58)]; *p* < 0.01.	Moderate
Jenkins et al., 2013, US [67]	Control site: Non: 21351. Intervention: 11619. Intervention site: Non: 10017. Intervention: 5403. Gender not mentioned	Not mentioned.	RCT	RTIs and UTIs.	**Developing clinical pathways for eight common adult and pediatric outpatient infections.**	Change over time in antibiotic prescriptions for non-pneumonia acute respiratory infections.	Trend of antibiotics used: Non: F(1, 35968) = 0.5, *p* = 0.49; Intervention: (F(1, 35968) = 66.9, *p* < 0.01.	High
						Change over time in broad-spectrum antibiotic prescriptions.	Trend of antibiotics used: Non: F(1, 48367) = 1.1; *p* = 0.29. Intervention: F(1, 48367) = 41.5, *p* <0.01.	
March-López et al., 2020, Spain [68]	260,561 (49.1)	Mean age (SD) 40.85 (22.81)	Interventional before and after study.	RTIs and UTIs.	**Multi-faceted intervention.** ASP presentation to all relevant stakeholders. Actions for improving antibiotic prescribing. Tracking and feedback. Guidelines’ implementation with physician education.	Overall antibiotic consumption [as defined daily doses per 1000 inhabitants per day (DID)].	Non: 16.01 DID Intervention: 13.31 DID	Serious
May et. al., 2021, USA [69]	Control site: Pre-intervention: 150 (64.0); Intervention: 150 (70.7). Intervention site: pre-intervention: 130 (61.5) Intervention: 99 (52.5); Post-intervention: 54 (51.9).	Mean (SD) Control site: Pre-intervention: 43.2 (18.6) Intervention: 39.6 (18.2) Intervention site: pre-intervention: 43.9 (18.1); Intervention: 42.0 (18.0); Post-intervention: 40.7 (19.2).	Interventional before and after study	SSTIs	**Multifaceted intervention** HCP education (guidelines) Guidelines implementation (through algorithms). Order sets implementation	Clinician adherence to guidelines.	**Number of patients Guideline’s adherence (%):** Control site: Pre: 29 (19%); post: 38 (25%); OR = 1.82 (95% CI 0.79, 4.21); (non-significant) Intervention site: Pre: 53 (41%); Post: 28 (51%); OR = 1.17 (95% CI 0.65, 2.12). (non-significant) Difference-in-differences Between sites of during vs. pre-intervention was not statistically significant [OR = 1.82 (95% CI 0.79, 4.21)].	Moderate
Slekovec et al., 2012. France [70]	Number of prescriptions Non-intervention: 2972 Intervention: 3279 All females	Aged 15–65	Interventional before and after study	UTIs	**Guidelines’ implementation:** Two main messages: 1-FQs should not be used for uncomplicated acute cystitis. 2- Fosfomycin or nitrofurantoin should be preferred as first-line treatment for uncomplicated UTIs.	Number of antibiotic prescriptions of nitrofurantoin, Fosfomycin-trometamol and fluoroquinolones.	Number of nitrofurantoin prescriptions: Non: 295.9 (279.5–312.4); Intervention: 398.9 (370.4–427.3); Increased by 36.8% (95% CI: 30.6, 42.2); *p* < 0.01. Number of Fosfomycin-trometamol prescriptions: Non: 1082.8 (95% CI 1011.2, 1154.5); Intervention: 1412.6 (95% CI 1344.0, 1481.2); Increased by 28.5% (95% CI: 22.9, 35.4); *p* < 0.01. Number of Norfloxacin prescriptions: Non: 836.9 (95% CI 800.5–873.4); Intervention: 737.5 (95% CI 703.3, 771.7); Decreased by 9.1% (95% CI: −15.3, −3.5); *p* < 0.01.	Moderate
Vinnard et. al., 2013, US [71]	Control group: Pre: 320; Post-Intervention: 320. Intervention group: Pre-Intervention: 254; Intervention: 392. Gender not mentioned	Adults	Interventional before and after study	RTIs	**HCP education** The intensive intervention group received academic detailing by a pharmacist and an opinion leader in antibiotic use. **Patient Education.**	The proportion of visits for acute bronchitis or URTIs for which there was prescription of at least 1 antibacterial antibiotic.	**Number of visiting patients (%):** Non-intervention: Pre: 191 (59.7%); Post: 186 (58.1%). Intervention: Pre: 60 (23.6%). Post: 50 (12.8%); *p* = 0.133.	Moderate

ITS: interrupted time series; RCT: randomized control trial; DOT: days of therapy; LOS: length of hospital stay; CI: confidence interval; SD: standard deviation; IQR: inter Quartile Range; HR: hazard ratio; OR: odds ratio; RR: risk ratio; IRR: Incidence rate ratio; ARR: adjusted relative risk; CDI: Clostridioides difficile infection; PD: patient days; ICU: intensive care unit; RTI: respiratory tract infection; UTI: urinary tract infections; SSTIs: skin and soft tissue infections; FQs: Fluoroquinolones; OBD: occupied bed days; USD: United States dollar; MRSA: methicillin resistant staphylococcus aureus; ESBL: extended spectrum beta lactamase; CRO: carbapenem resistant organisms; AMU: antimicrobial use; ASP: antimicrobial stewardship program; DID: daily defined dose per 1000 inhabitants-day; DDD: daily defined dose.

**Table 2:** Types of intervention per setting.

Intervention/Setting	Studies
**IP with a pharmacist as part of the antimicrobial stewardship team**	
Pocket cards containing antimicrobial guidelines.	[43]
Prospective audit and feedback with direct intervention.	[37,38,42,44,45,47,48,49,50,53,54]
IV to oral guideline implementation.	[41]
Antimicrobial treatment guidelines’ implementation.	[51]
Clinical decision support system use.	[46]
Implementation of antibiotic time out.	[52]
HCP education.	[40]
Prospective audit and feedback.	[40]
MDT escalating approach.	[55]
**IP without a pharmacist as part of the antimicrobial stewardship team**	
Soft stop order.	[31]
Clinical decision support system use.	[31,39]
Antibiotic restriction and pre-authorization.	[24,30,33]
HCP education.	[25,28]
Regular dedicated IDT rounds.	[27,29,34]
Prospective audit and feedback with direct intervention.	[32,44,53]
Clinical decision-making algorithm.	[36]
Antimicrobial treatment guidelines’ implementation.	[35]
Multi-faceted IDT visits (rounds, interactive training sessions, meetings)	[26]
**OP with a pharmacist as part of the antimicrobial stewardship team**	
HCP education after audit and feedback.	[64]
HCP education.	[66,68,69,71]
Patient education.	[71]
Antimicrobial treatment guideline implementation.	[69,70]
Order sets implementation.	[69]
Retrospective audit and feedback.	[65,66]
Developing clinical pathways for common OP infections.	[67]
Antimicrobial guidelines’ implementation with physician education.	[68]
Clinical decision support system use.	[66]
**OP without a pharmacist as part of the antimicrobial stewardship team**	
Prospective audit and feedback.	[62]
Antimicrobial treatment guidelines’ implementation.	[62]
Shared decision making.	[58]
Creating quality dashboard.	[59]
Antibiotic restriction and pre-authorization.	[61]
HCP education.	[57]
Delayed antibiotic prescriptions.	[60]

OP: outpatient setting; IP: inpatient setting; HCP: health care professional; IDT: infectious disease team.

**Table 3:** Search Strategy.

Database	Search Within	Number of Results	Key Words
**PUBMD**	All fields (Filter: Clinical trials only, period 2010–2022, English only)	154	**P** (hospital OR hospitals OR inpatient OR inpatients OR outpatients OR outpatient OR primary care) AND **I**: ((antibiotic stewardship) OR (antimicrobial stewardship) OR (antibacterial stewardship)) AND **O**: (outcome OR outcomes OR use OR utilization OR implementation OR prescribing OR prescription OR consumption OR mortality OR hospital stay OR therapy days OR difficile OR MDR OR MRSA OR ESBL OR Appropriate OR infection OR infections).
**CINAHL**	All fields (Filter: Academic journals, period 2010–2022, All adults, English only)	351	
**SCOPUS**	Titles, abstracts, keywords. (Filter: Medicine, Article, Final, Journal, English only).	1551	

## Data Availability

Data were analyzed and presented in this paper.

## Supplementary Materials

The following supporting information can be downloaded at: https://www.mdpi.com/article/10.3390/antibiotics11101306/s1, Figure S1: Funnel plot assessing the risk of publication bias for mortality; Figure S2: Funnel plot assessing the risk of publication bias for readmission rate; Figure S3: Funnel plot assessing the risk of publication bias for the prevalence of antibiotics prescribed; Figure S4: Funnel plot assessing the risk of publication bias for the percentage of adherence to antimicrobial guidelines.
