# Supplementary material for: Exploring Antimicrobial Stewardship Influential Interventions on Improving Antibiotic Utilization in Outpatient and Inpatient Settings: A Systematic Review and Meta-Analysis
Source: Antibiotics (Basel). 2022 Sep 26;11(10):1306. doi: 10.3390/antibiotics11101306 (PMC9598859; doi:10.3390/antibiotics11101306)
Supplement: Supplementary file 1 [file antibiotics-11-01306-s001.zip › antibiotics-1908354-supplementary.pdf]

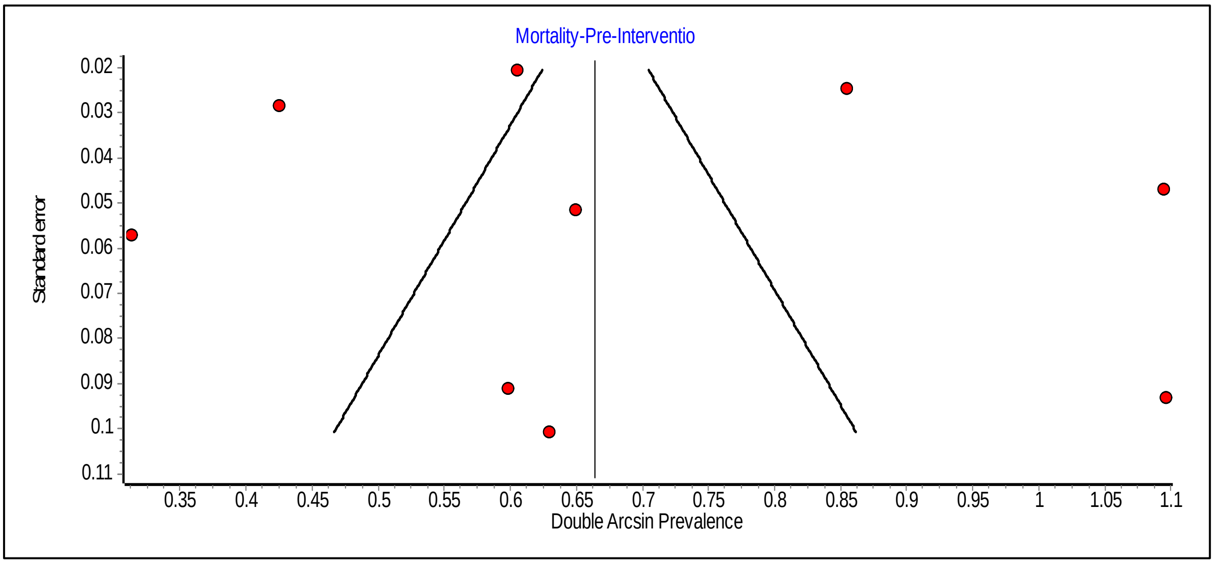

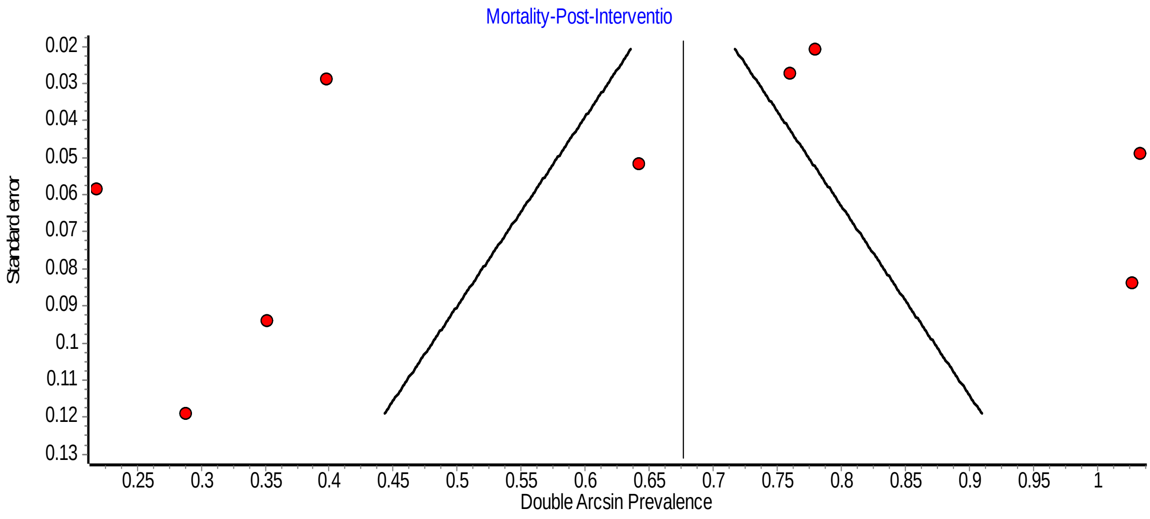

Figure S1: Funnel plot assessing the risk of publication bias for mortality.

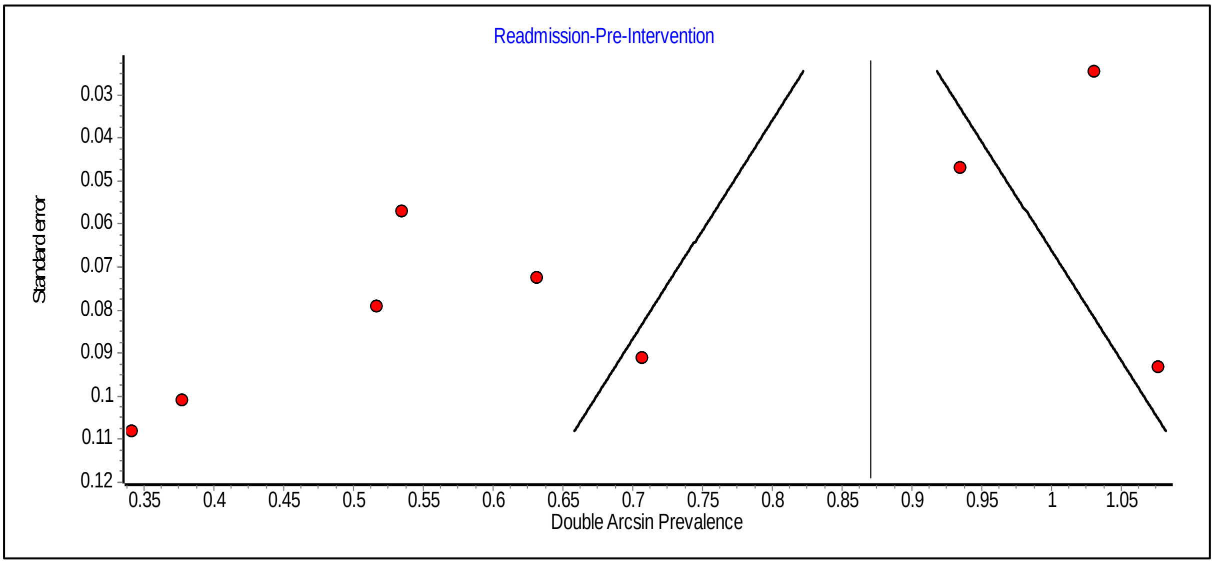

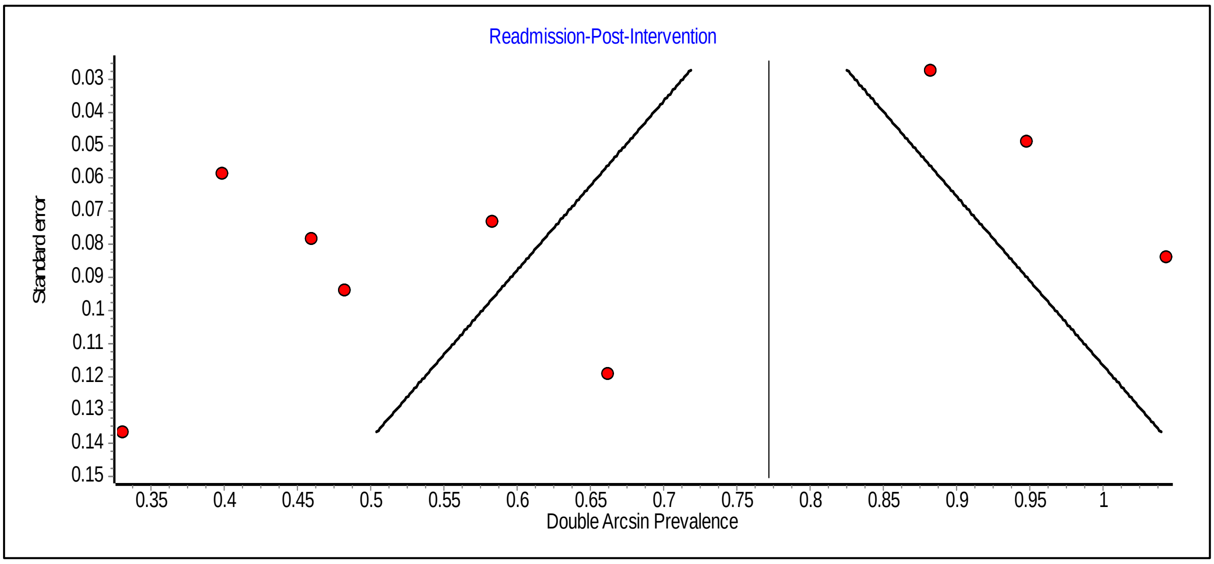

Figure S2: Funnel plot assessing the risk of publication bias for readmission rate.

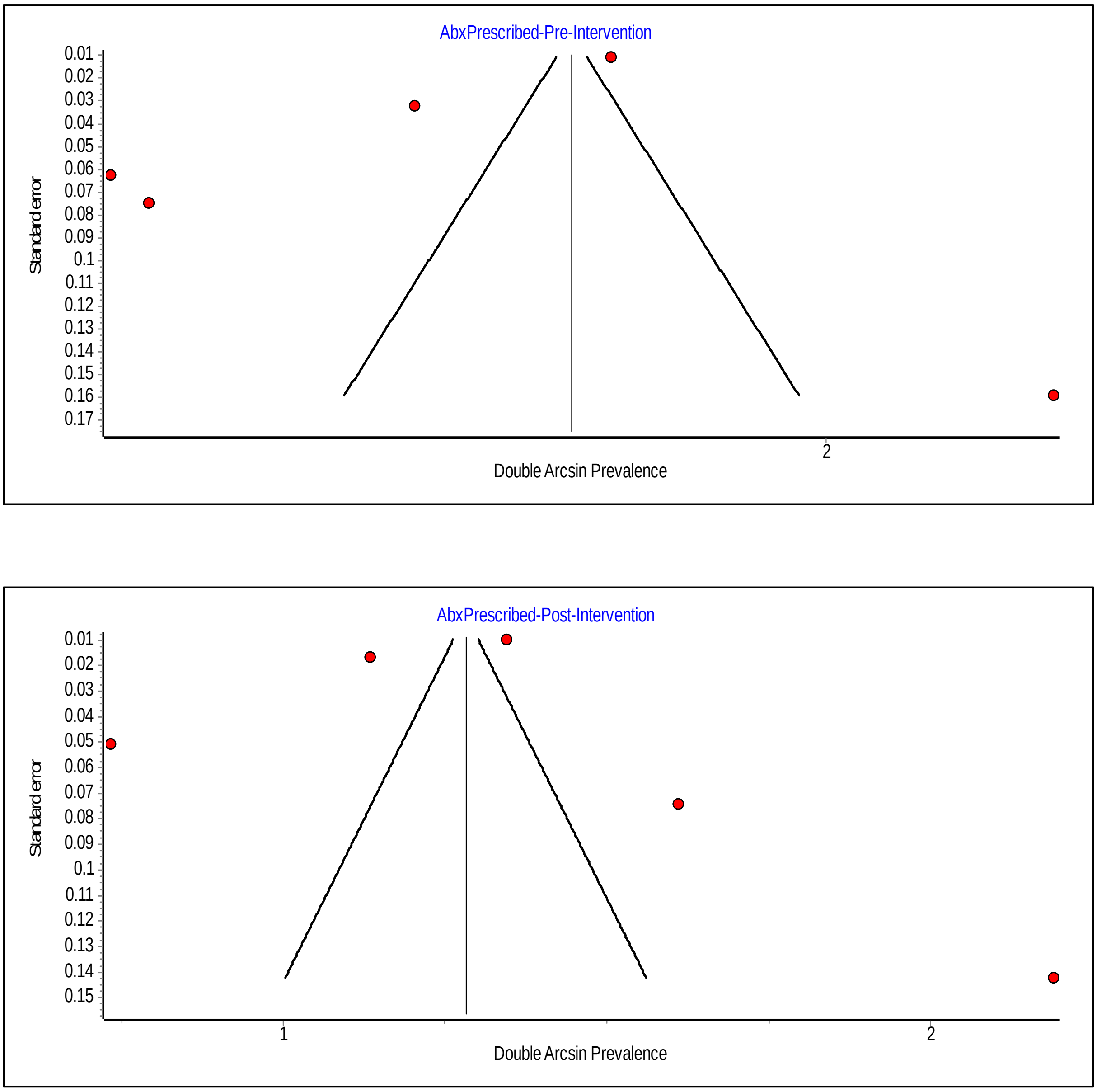

Figure S3: Funnel plot assessing the risk of publication bias for the prevalence of antibiotics prescribed.

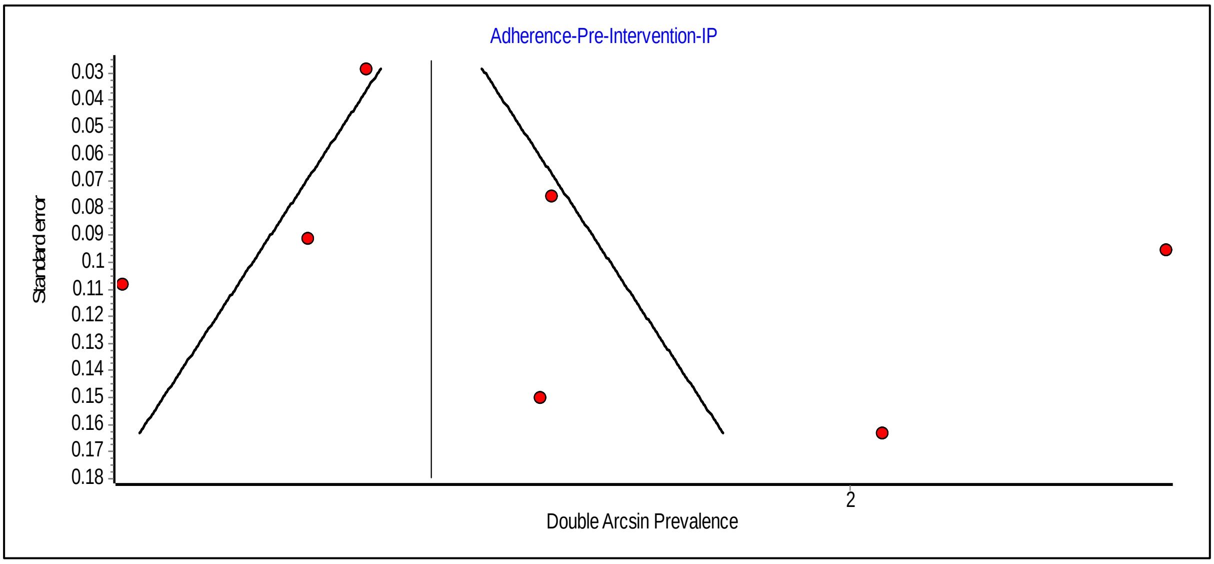

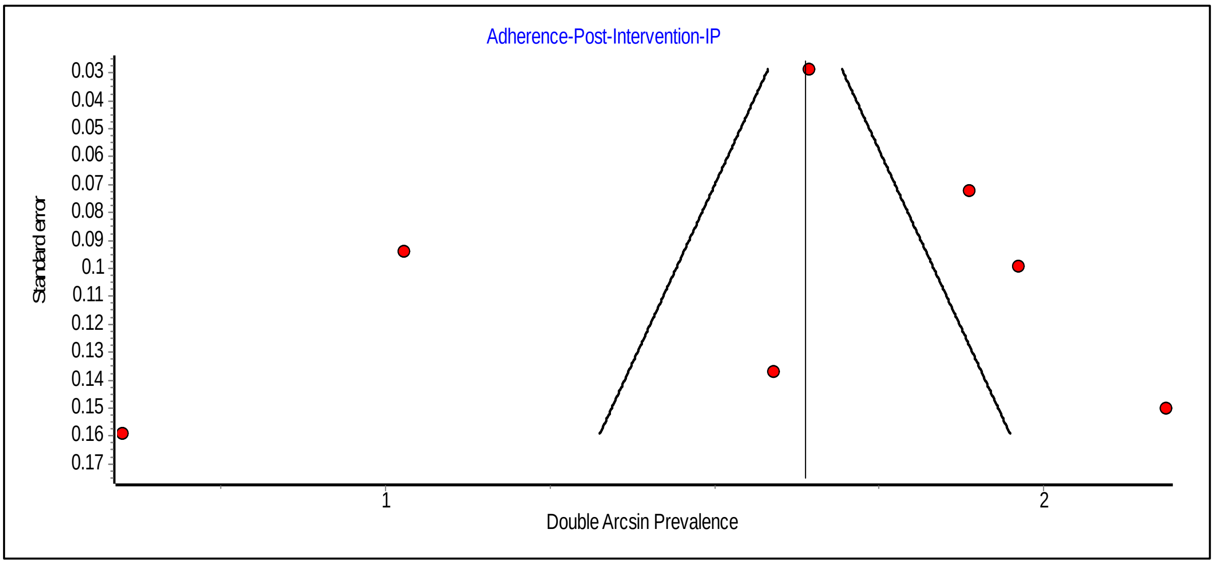

Figure S4: Funnel plot assessing the risk of publication bias for the percentage of adherence to antimicrobial guidelines.
